# Divergent roles of IL-35 and IL-39 in rheumatoid arthritis: restoring cytokine balance within the IL-12 family

**DOI:** 10.3389/fimmu.2026.1810466

**Published:** 2026-04-13

**Authors:** Xingyan Ling, Xuhui Zhang, Jieliang Hu, Brett D Hambly, Shisan Bao

**Affiliations:** 1 Scientific Research Section, The Third Affiliated Hospital of Gansu University of Chinese Medicine, Baiyin, China; 2 Respiratory Centre, The Third Affiliated Hospital of Gansu University of Chinese Medicine, Baiyin, China; 3 Joint Surgery, The First People’s Hospital of Baiyin, The Third Affiliated Hospital of Gansu University of Chinese Medicine, Baiyin, China

**Keywords:** divergent role, IL-35, IL-39, rheumatoid arthritis, therapeutic potential

## Abstract

Rheumatoid arthritis (RA) is a chronic autoimmune disease characterised by persistent synovial inflammation, progressive cartilage destruction, and irreversible bone erosion. Although substantial progress has been made in identifying downstream inflammatory mediators, the upstream regulatory architecture governing immune imbalance in RA remains incompletely understood. Members of the interleukin-12 (IL-12) cytokine family are key regulators of T-cell differentiation and inflammatory amplification. Among them, IL-35 and IL-39 represent functionally opposing yet incompletely characterised cytokines with emerging relevance to RA pathogenesis. IL-35, predominantly produced by regulatory T and B cells, exerts immunosuppressive effects by inhibiting T helper 17 (Th17) responses, expanding regulatory lymphocyte populations, and modulating macrophage polarisation. Evidence suggests dysregulation of the IL-35 axis in RA, characterised by reduced systemic levels but relative synovial upregulation, possibly reflecting a compensatory response to persistent inflammation. In contrast, IL-39, derived from activated B cells and myeloid cells, promotes inflammatory cascades through STAT1/STAT3 signalling. Circulating IL-39 levels correlate with disease activity and inflammatory biomarkers, supporting its potential role in sustaining immune activation. This mini-review synthesises current evidence on the divergent immunobiology of IL-35 and IL-39 in RA, evaluates their mechanistic pathways, and discusses their translational implications as biomarkers and therapeutic targets. By examining IL-35 alongside the relatively understudied cytokine IL-39, we highlight the added value of this pairing in clarifying their shared and distinct biological functions. We propose that disruption of regulatory–inflammatory equilibrium within the IL-12 cytokine family provides a conceptual framework for understanding immune dysregulation in RA and may inform precision immunomodulatory strategies.

## Introduction

Rheumatoid arthritis (RA) is a chronic systemic autoimmune disease characterised by persistent synovial inflammation, progressive cartilage destruction, and bone erosion, most commonly affecting the small joints of the hands and knees (1). Although the precise molecular mechanisms underlying RA remain incompletely understood (2), substantial evidence indicates that the disease arises from a breakdown of immune homeostasis, resulting in a sustained imbalance between pro-inflammatory and anti-inflammatory pathways (3). This imbalance reflects coordinated dysregulation of both innate and adaptive immune compartments, culminating in chronic synovitis and systemic inflammatory involvement (4).

Genetic susceptibility, particularly involving major histocompatibility complex class II DR beta 1 (HLA-DRB1) alleles, provides a permissive background for aberrant immune activation (5). In predisposed individuals, excessive activation of innate immune cells, especially macrophages skewed toward the pro-inflammatory M1 phenotype, drives overproduction of tumour necrosis factor (TNF), IL-6, and IL-1β, amplifying synovial inflammation (6). Concurrently, adaptive immune imbalance sustains disease progression. Expansion of T helper 17 (Th17) cells and impairment of regulatory T cells (Tregs) disrupt immune tolerance and reinforce inflammatory signalling (7). Th17-derived IL-17 promotes neutrophil recruitment, synovial fibroblast activation, and matrix degradation (8, 9), whereas insufficient Treg-mediated suppression fails to restore immune equilibrium. These interconnected abnormalities establish a self-perpetuating inflammatory circuit driving pannus formation, osteoclast activation, and structural joint damage (2).

RA is increasingly recognised as a systemic inflammatory disorder rather than a disease confined to the joints. Persistent cytokinaemia, autoantibody production, and immune complex deposition contribute to extra-articular manifestations, including interstitial lung disease and increased cardiovascular risk (3, 10). Thus, RA pathogenesis is governed by dynamic cytokine networks operating at both local and systemic levels (8).

While current biologic therapies primarily target dominant pro-inflammatory mediators such as TNF and IL-6, upstream cytokines that shape immune balance remain less well characterised (6). The roles of IL-1 family members, including IL-36, IL-37, and IL-38, in RA have been recently reviewed (11, 12). Similarly, IL-12 family cytokines, including IL-12, IL-23, IL-27, and IL-35, have been extensively studied in autoimmune diseases such as systemic lupus erythematosus, systemic sclerosis, psoriasis, inflammatory bowel disease, and Sjögren’s syndrome (13). These cytokines have emerged as critical modulators of T-cell differentiation and macrophage programming, highlighting their therapeutic potential.

Within the IL-12 cytokine family, IL-35 and IL-39 share the Epstein-Barr virus-induced gene 3 (EBI3) subunit but display opposing immunological functions, due to their partner subunit, p35 (IL-12A) in IL-35 and IL-23p19 in IL-39 (14).

In periodontitis, IL-35 levels in gingival crevicular fluid are reduced, particularly in patients with type 2 diabetes mellitus (T2DM), but increase following non-surgical periodontal therapy (15), whereas IL-39 is elevated and declines after treatment. These findings suggest opposing roles, with IL-35 contributing to local anti-inflammatory responses and IL-39 promoting inflammation, although mechanistic pathways and systemic expression remain unclear. A comparable context-dependent pattern is observed in Brucellosis, where both cytokines are suppressed during the acute phase but become upregulated in chronic disease, exhibiting a positive correlation (16). This likely reflects persistent intracellular infection driving sustained immune activation, in which IL-39 contributes to ongoing inflammation, while IL-35 is concurrently induced as a compensatory immunoregulatory mechanism, primarily via regulatory T and B cells, to limit immune-mediated damage (17). Mechanistically, IL-35 signalling through STAT1/STAT3 promotes the expansion of regulatory T-cell populations and suppresses Th17 responses, thereby reinforcing negative feedback control of inflammation (18). Accordingly, their positive correlation may represent a dynamic equilibrium between inflammatory and regulatory responses under chronic stimulation.

In contrast, in localised conditions such as Gingivitis, immune responses are predominantly innate and may lack sufficient magnitude or duration to induce robust regulatory cytokine production. Moreover, the gingival microenvironment, shaped by bacterial biofilms and pathogen-associated molecular patterns, preferentially sustains pro-inflammatory signalling while impairing regulatory pathways, thereby limiting IL-35 upregulation (19). Collectively, these observations underscore that IL-35 and IL-39 expression is highly context-dependent, reflecting the balance between inflammatory burden and the capacity to mount effective immunoregulatory feedback.

These observations highlight that IL-35 and IL-39 exert context-dependent, often opposing roles in inflammation. Although these findings cannot be directly extrapolated across diseases, they provide a rationale for investigating whether similar regulatory dynamics occur in RA, where dysregulated local and systemic inflammation may be influenced by IL-35 and IL-39. A key limitation of the studies discussed above is the lack of mechanistic analyses, leaving the regulatory dynamics of these cytokines incompletely understood.

Current evidence indicates that IL-35 primarily exerts immunoregulatory (20, 21) and anti-inflammatory effects, whereas IL-39 appears to promote inflammatory responses. These contrasting functions arise from their distinct partnering subunits - p35 (IL-12A) in IL-35 and IL-23p19 in IL-39. Examining these cytokines together therefore provides insight into how two EBI3-containing cytokines can mediate opposing immunological effects within a shared signalling framework. While other members of the IL-12 cytokine family, such as IL-12 (22) and IL-23 (23), have been well characterised in RA, IL-39 remains comparatively understudied.

Although IL-35 has been widely reviewed, the present work situates it alongside IL-39, emphasising the added value of pairing it with this relatively understudied cytokine to highlight their shared and distinct biological functions that have not been systematically addressed. Examining these cytokines together allows a focused mini-review to provide mechanistic and translational insight into immune balance, exploring how dysregulation of these axes may contribute to disease pathogenesis.

In this mini-review, we critically examine the molecular architecture, signalling pathways, and compartment-specific expression patterns of IL-35 and IL-39 in RA. We further discuss their implications for biomarker development and targeted immunomodulatory strategies, highlighting how understanding these complementary cytokines may inform pathway-specific precision immunotherapy.

## IL-35

IL-35 is a heterodimeric cytokine of the IL-12 family composed of the p35 (IL-12A) and EBI3 subunits, primarily produced by regulatory T (Treg) and regulatory B (Breg) cells (24, 25). It functions as a potent immunosuppressive mediator that limits excessive immune activation and maintains immune homeostasis.

IL-35 signals through receptor complexes involving IL-12Rβ2 and gp130, which may form heterodimeric or homodimeric pairings depending on the cellular context. Receptor engagement activates Janus kinase (JAK)/signal transducer and activator of transcription (STAT) pathways. In T cells, IL-35 predominantly induces STAT1 and STAT4 activation, whereas in B cells it promotes phosphorylation of STAT1 and STAT3. Functionally, IL-35 suppresses effector T-cell proliferation, inhibits Th1 and Th17 differentiation, and promotes the expansion and stabilisation of Treg and Breg populations (26). It also promotes macrophage polarisation towards an anti-inflammatory M2 phenotype (27), partly through STAT-dependent mechanisms and suppression of nuclear factor κB (NF-κB)-mediated transcription of pro-inflammatory genes.

It should be noted that much of this mechanistic evidence derives from experimental and non-RA inflammatory models. Direct validation in human RA synovial tissue remains limited, and extrapolation to RA pathogenesis should therefore be interpreted cautiously.

Preclinical studies consistently demonstrate protective effects of IL-35 in autoimmune and inflammatory settings. In experimental arthritis, IL-35 attenuates joint inflammation and tissue destruction (28), while in cardiovascular models it exerts anti-inflammatory and vasculoprotective actions (21). These effects are largely mediated through STAT1/STAT4 signalling and downregulation of pro-inflammatory cytokines, including TNF, IL-6, and IL-17 (29). Collectively, IL-35 appears to function as a regulatory node bridging innate and adaptive immunity, although its precise contribution to RA in humans remains incompletely defined.

## IL-35 in RA

Elevated circulating IL-35 levels have been reported in several cohorts of patients with RA compared with healthy controls, with associations observed between IL-35 concentrations and established clinical indices, including erythrocyte sedimentation rate (ESR), C-reactive protein (CRP), global health on the visual analogue scale, Disease Activity Score using 28 joint counts (DAS28-ESR), rheumatoid factor (RF), and anti-citrullinated protein antibodies (ACPAs) (30). Notably, multivariate analyses indicate that higher serum IL-35 levels correlate with lower ESR and DAS28-ESR (30, 31), suggesting that IL-35 may reflect activation of endogenous regulatory pathways rather than simply mirroring inflammatory burden.

Consistent with this interpretation, synovial fluid IL-35 concentrations are also elevated in RA compared with osteoarthritis controls (32). Although osteoarthritis represents an imperfect comparator and sample sizes were modest, the compartment-specific increase of IL-35 within inflamed joints supports its involvement in the local immunological milieu. However, the persistence of synovitis despite increased IL-35 levels suggests that this response is either quantitatively insufficient or functionally impaired to restore immune homeostasis.

Mechanistically, such a compensatory interpretation is plausible. The RA synovium is enriched in pro-inflammatory M1 macrophages producing TNF, IL-6, and IL-1β (6). In other inflammatory contexts, IL-35 promotes M2 macrophage polarisation and enhances regulatory T-cell activity (33). Within RA, therefore, IL-35 upregulation may represent an endogenous attempt to counterbalance macrophage-driven inflammation and Th17-mediated pathology. The inability of this compensatory axis to fully suppress disease progression suggests a breakdown of regulatory dominance, positioning IL-35 as a protective but functionally overwhelmed mediator within the inflammatory network.

Preclinical data further support this protective model. In collagen-induced arthritis (CIA), administration of exogenous IL-35 reduces histopathological severity, diminishes leukocyte infiltration and synovial hyperplasia, and limits cartilage and bone erosion (28). These improvements are accompanied by expansion of regulatory T-cell populations, suppression of Th17 responses, and increased IL-10 production (20). Although CIA does not fully recapitulate human RA, these findings provide proof-of-concept that enhancing IL-35.

Nevertheless, clinical observations are not entirely consistent. Some studies report reduced circulating IL-35 levels in patients with active RA, together with inverse correlations between IL-35 concentrations and DAS28-ESR, CRP, ESR, and RF (34, 35). Patients in remission, by contrast, exhibit relatively higher IL-35 levels. These findings may still support the protective hypothesis: insufficient IL-35 availability could remove an important regulatory brake, thereby permitting sustained inflammatory activation.

The discrepancies observed across studies may partly reflect genetic heterogeneity. Existing cohorts originate from diverse ethnic and geographic backgrounds (30, 31, 34, 35), and associations between IL-35 gene polymorphisms and RA susceptibility have been described (31). Such genetic variation may influence IL-35 production, receptor signalling efficiency, or downstream regulatory capacity, highlighting the need for stratified longitudinal studies across different populations.

From a therapeutic perspective, current RA management largely relies on disease-modifying antirheumatic drugs (DMARDs), including Abatacept (1, 8) and biologic agents targeting TNF, the IL-6 receptor, or co-stimulatory pathways (36). While these therapies effectively suppress inflammatory cascades, they do not specifically restore endogenous regulatory circuits and may carry long-term safety concerns (37). In contrast, therapeutic augmentation of IL-35 signalling represents a conceptually distinct approach aimed at reinforcing intrinsic immunological restraint.

Collectively, the available data suggest that IL-35 functions primarily as an endogenous immunoregulatory cytokine that attempts to counterbalance inflammatory responses in RA. However, the IL-12 cytokine family encompasses additional members with potentially distinct and less well-characterised functions. Among these, IL-39 has recently emerged as a candidate inflammatory mediator, although its biological existence, signalling mechanisms, and relevance to autoimmune diseases remain the subject of ongoing debate (38).

## IL-39

Bridgewood and colleagues describe that the proposed IL-39 cytokine consists of the IL-23p19 subunit (shared with IL-23) and the EBI3 subunit (shared with IL-27 and IL-35), based on heterodimer formation in the IL-12 cytokine family, although its existence in humans remains unconfirmed experimentally (39). It is primarily produced by activated B cells, dendritic cells, and macrophages and has been proposed as a pro-inflammatory cytokine. While IL-39 has been clearly demonstrated to have an established role in mice (40), several studies over the last decade have questioned its existence and functionality in humans (39, 41). One study failed to detect IL-39 in tissue culture supernatant from stimulated human immune cells and keratinocytes, and found that recombinant human IL-39 did not stimulate human cells (41). On the other hand, several studies, detailed below, have detected IL-39 in human serum using various commercially available antisera, although the precise epitopes utilised by these antisera are generally not specified by the manufacturers, implying that these antisera may be detecting other members of the IL-12 family containing one or other of the IL-39 subunits. This question needs to be clarified, primarily by co-immunoprecipitation of human IL-39 derived from elevated levels believed to be present in human disease states, and the subsequent use of this human-derived IL-39 to functionally stimulate relevant human immune cells.

In lupus-like murine models, IL-39 drives systemic inflammation and accelerates disease progression, whereas genetic or functional depletion of either p19 or EBI3 attenuates splenomegaly and proteinuria, supporting a pathogenic role in immune-mediated disorders. Mechanistically, IL-39 signals through a receptor complex composed of IL-23R and gp130, leading to activation of STAT1 and STAT3 pathways (40). These signalling cascades enhance inflammatory gene transcription and amplify immune activation.

Beyond experimental models, elevated circulating IL-39 levels have been reported in type 2 diabetes mellitus, correlating with neuropathy and increased body mass index, suggesting involvement in chronic systemic inflammation (42). Increased IL-39 expression has also been observed in inflammatory bowel disease (43) and several malignancies, including bladder and pancreatic cancer (44). Collectively, these observations position IL-39 as a mediator linking metabolic dysregulation, chronic inflammation, and tumour-associated immune imbalance.

## IL-39 in rheumatoid arthritis: emerging evidence and unresolved questions

Clinical evidence suggests that IL-39 may be elevated in RA. In a cohort of Chinese patients, circulating IL-39 concentrations were significantly higher than in non-RA controls and positively correlated with disease activity parameters, including DAS28, ESR, rheumatoid factor, IgM, and C-reactive protein (45). Receiver operating characteristic analyses indicated favourable sensitivity and specificity, suggesting potential diagnostic and disease-monitoring value. These data imply that IL-39 mediates dysregulated host immunity, contributing to RA pathogenesis.

Since mechanistic data defining IL-39’s precise role in RA remains limited, insights may be drawn from other inflammatory conditions. In patients with active ulcerative colitis and Crohn’s disease, IL-39 expression at both mRNA and protein levels is markedly elevated across affected intestinal tissue compared with individuals in remission and healthy controls (43). This local upregulation is accompanied by increased circulating IL-39 levels, and higher tissue expression correlates with histopathological severity scores. Collectively, these observations suggest that IL-39 functions as an important inflammatory mediator in chronic inflammatory diseases.

Extrapolating from these findings, IL-39 may contribute to RA development, particularly in genetically susceptible individuals carrying HLA-DRB1 alleles (5), potentially through STAT1/STAT3 signalling pathways, as suggested by lipopolysaccharide induced animal models (40). These observations imply that IL-39 may participate in both local and systemic inflammatory responses and could represent a potential therapeutic target. However, such translational implications should be interpreted cautiously. Critical issues, including the long-term efficacy of antibody-based interventions, potential anti-drug immune responses, and the risk of off-target or organ-specific toxicity, require careful evaluation before IL-39-targeted strategies can be considered clinically viable.

Independent studies in Iranian RA cohorts also report increased circulating IL-39 levels in treatment-naïve patients, individuals receiving conventional DMARDs, and those treated with anti-TNF agents (46). Notably, IL-39 elevation appears independent of anti-cyclic citrullinated peptide (anti-CCP) serostatus, suggesting that its upregulation may represent a core inflammatory feature of RA rather than a serotype-restricted phenomenon. The persistence of elevated IL-39 despite anti-TNF therapy further implies incomplete suppression of upstream inflammatory pathways. However, data on local IL-39 expression within synovial tissue remains to be determined, and no correlations have yet been established between IL-39 levels and joint disease severity at either the local or systemic level. These gaps highlight the importance of further investigation and underscore the importance of developing more effective therapeutic strategies.

Despite these findings, several limitations constrain interpretation. IL-39 expression within synovial tissue remains undefined, and the relationship between local IL-39 expression and joint pathology has not yet been evaluated using imaging or synovial biopsy - approaches previously applied in studies of IL-38 in RA (47). Consequently, it remains unclear whether circulating IL-39 accurately reflects local synovial inflammation, systemic immune activation, or both. More importantly, IL-39 mRNA and/or protein expression in RA-affected joints has not yet been determined; therefore, the observations described above remain preliminary and insufficient to establish a definitive role for IL-39 in RA pathogenesis. Furthermore, the underlying signalling pathways, such as STAT1/STAT3 activation, and potential downstream targets within damaged joints remain to be clarified.

Functional validation of IL-39 is still absent. Direct *in vitro* studies examining the effects of recombinant IL-39 on RA-derived immune cells, such as macrophages, together with investigations using genetically modified arthritis models, are required to determine whether IL-39 plays a causal role rather than merely being associated with disease activity (48). Furthermore, although IL-39 has been proposed as a novel member of the IL-12 cytokine family, its biological existence, receptor composition, and reproducibility remain subjects of debate. Independent validation across laboratories will therefore be necessary to clarify its biological relevance.

For example, although IL-39 has been shown to be produced by activated B cells and neutrophils and to mediate inflammation in lupus-like mice (40), recombinant human IL-39 fails to induce pro-inflammatory cytokine production or STAT3 phosphorylation in human PBMCs *in vitro*, leading some investigators to regard it as a “theoretical cytokine” (39). Nevertheless, antibody blockade in a very small phase II clinical trial demonstrated some promising outcomes in psoriasis patients (39). These apparently contradictory findings highlight the ongoing uncertainty surrounding IL-39 biology and underscore the need for caution when considering therapeutic manipulation of this cytokine. Importantly, this uncertainty contrasts with the comparatively better-characterised immunoregulatory functions of IL-35. Clarifying the biological validity, signalling mechanisms, and disease relevance of IL-39 will therefore be essential to determine whether it represents a genuine cytokine involved in RA pathogenesis or merely a theoretical construct within the broader IL-12 cytokine network.

Despite both IL-35 and IL-39 activating STAT1 and STAT3 pathways, their functional outcomes diverge substantially due to differences in receptor composition, STAT dimerisation, and cellular context. IL-35 signals through IL-12Rβ2 and gp130 heterodimers, promoting STAT1–STAT3 and STAT1–STAT4 interactions that drive anti-inflammatory transcriptional programmes, including the expansion of regulatory T-cell populations and suppression of Th17 responses (49). In contrast, IL-39 is proposed to preferentially induce STAT3-dominant signalling, particularly in innate immune cells, thereby amplifying inflammatory cascades (40). These findings highlight that shared STAT activation does not equate to shared function; rather, signalling specificity is determined by receptor context and downstream transcriptional networks.

**Figure 1:**
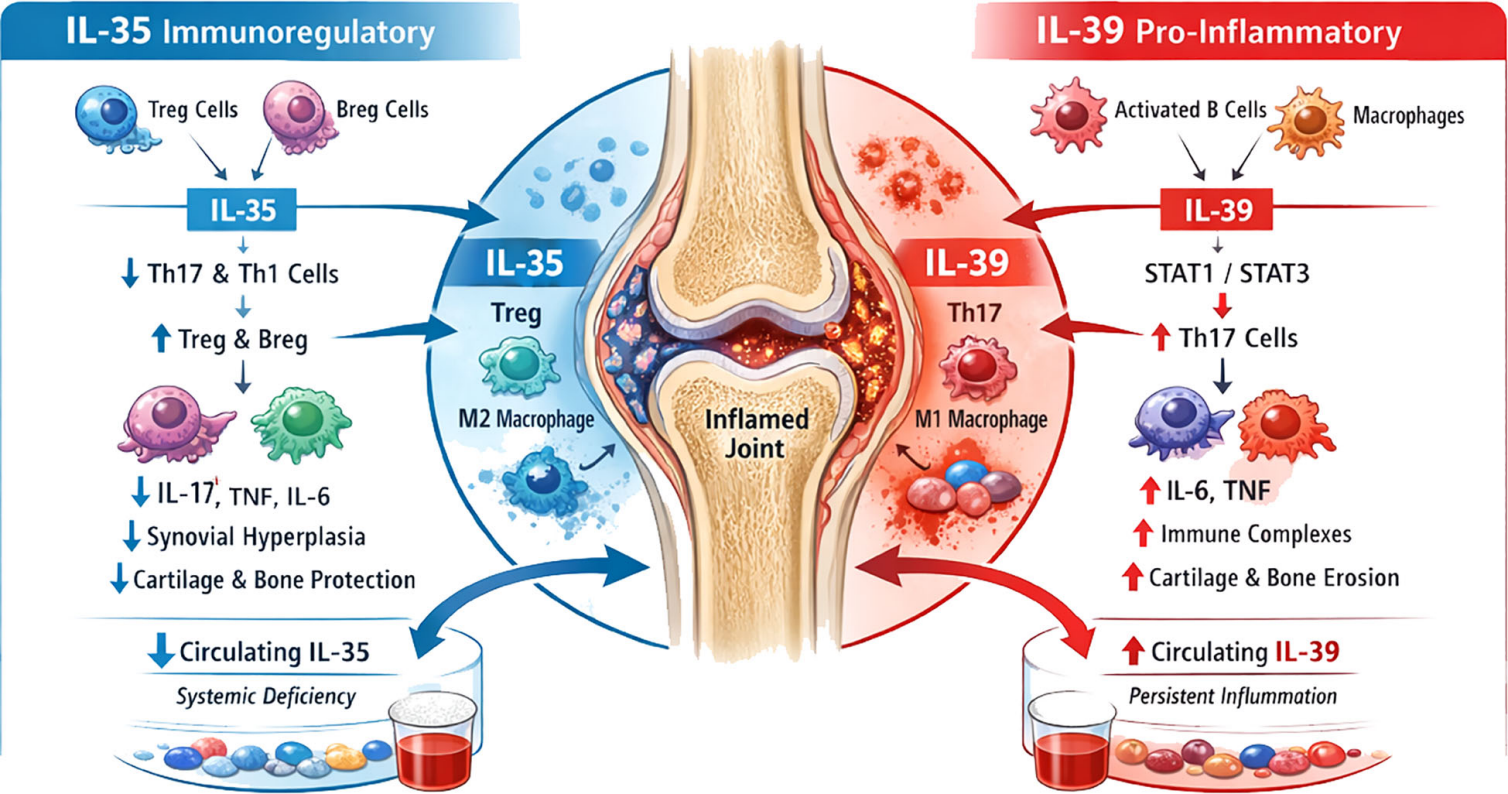
Opposing roles of IL-35 and IL-39 in RA. IL-35, produced by regulatory T (Treg) and B (Breg) cells, functions as an immunological brake. It signals via IL-12Rβ2/gp130 complexes, activating STAT1/STAT4 in T cells and STAT1/STAT3 in B cells, to suppress Th1 and Th17 responses, expand regulatory populations, and polarise macrophages towards an anti-inflammatory M2 phenotype. Within inflamed synovium, IL-35 may act as a local compensatory mechanism. In contrast, IL-39, derived from activated B cells, dendritic cells, and macrophages, acts as an inflammatory accelerator. It signals through IL-23R/gp130 to activate STAT1/STAT3, amplifying Th17-driven inflammation, enhancing M1 macrophage activity, and promoting pro-inflammatory cytokine production, thereby driving joint damage. These opposing axes illustrate compartment-specific immune regulation in RA, highlighting IL-35 as a regulatory brake and IL-39 as a pro-inflammatory accelerator. Both cytokines represent potential biomarkers and therapeutic targets, although their precise receptor usage and downstream signalling pathways require further clarification.

Taken together, the available evidence suggests that IL-35 and IL-39 may represent functionally divergent members of the IL-12 cytokine family in RA. IL-35 functions predominantly as an endogenous immunoregulatory mediator attempting to restrain inflammatory responses, although its compensatory activity may be insufficient to fully restore immune homeostasis during chronic disease. In contrast, the biological relevance of IL-39 remains far less certain, with limited and sometimes contradictory findings regarding its existence, signalling mechanisms, and pathogenic contribution to inflammatory disorders (**Figure 1**). Clarifying the interplay between these cytokines - including their cellular sources, receptor composition, and downstream signalling pathways - will therefore be essential for understanding how regulatory and pro-inflammatory axes within the IL-12 cytokine family influence RA pathogenesis. Such insights may ultimately help identify novel biomarkers and inform future cytokine-targeted therapeutic strategies.

## Future perspectives

Future studies should move beyond descriptive profiling towards mechanistic and spatially resolved analyses of IL-35 and IL-39 in RA, defining their cellular sources, receptor usage, downstream signalling pathways, and interactions with Th17 cells, regulatory T cells, B cells, and macrophages. Therapeutically, restoring immune balance rather than broadly suppressing inflammation represents a rational objective. Enhancing IL-35 signalling or selectively inhibiting IL-39 may provide targeted immunomodulatory strategies; however, their pleiotropic and context-dependent effects require rigorous preclinical validation, comprehensive safety assessment, and careful patient stratification.

Caution is warranted, as the roles of IL-35 - and particularly IL-39 - in RA remain controversial given their relatively recent characterisation, with evidence suggesting the potential to both promote and inhibit disease, as well as the possibility of unforeseen adverse effects. Integrating cytokine biology with clinical and molecular phenotyping may ultimately facilitate precision immunotherapy, shifting RA management from generalised immunosuppression towards pathway-specific immune recalibration.

Therapeutically, these pathways are highly relevant in RA, where aberrant cytokine signalling converges on the JAK–STAT axis. Janus kinase inhibitors effectively suppress inflammation by blocking STAT activation downstream of multiple cytokines, but this broad mechanism may also attenuate endogenous regulatory signals, including those mediated by IL-35 (50). In contrast, abatacept modulates T-cell co-stimulation by binding to CD80/CD86 on antigen-presenting cells and blocking CD28-mediated activation of T cells, thereby suppressing pathogenic T-cell responses and promoting immune homeostasis as a strategy that restores immune balance rather than globally suppressing cytokine signalling (51). Collectively, these observations highlight the importance of selectively targeting inflammatory pathways while preserving regulatory mechanisms, which may be critical for achieving optimal therapeutic outcomes.
